# COVID-19 vaccine acceptance and associated factors among parents of children between 6 and 12 years: a multicenter mixed method study in India

**DOI:** 10.3389/fpubh.2025.1513419

**Published:** 2025-06-25

**Authors:** Manisha Ghate, Kangjam Rekhadevi, Abhik Sen, Saurabh Sharma, Pallavi Shidhaye, Saritha Nair, Ashish Kumar, Pournami Jayesh, Sandip Patil, Shraddha Gurav, Sumit Aggarwal

**Affiliations:** ^1^National AIDS Research Institute, Pune, India; ^2^Regional Medical Research Centre (ICMR), Dibrugarh, India; ^3^Rajendra Memorial Research Institute of Medical Sciences, Patna, India; ^4^Indian Council of Medical Research (ICMR), New Delhi, India; ^5^National AIDS Research Institute (ICMR), Pune, India

**Keywords:** vaccine acceptance, parents, COVID-19 vaccine, mixed methods, India

## Abstract

**Background:**

It is well recognized that parents play a central role in making decisions for their children. Understanding willingness of parents to vaccinate their children against COVID-19 is important as it helps to develop effective strategies for maximizing vaccination coverage. We aimed to evaluate parental acceptance regarding COVID-19 vaccination for children between 6 and 12 years of age in India.

**Methods:**

A mixed-method study (March–September 2023) employed a structured questionnaire and in-depth interviews with parents of school-going and non-school-going children across five purposively selected Indian states. Multistage random sampling was used for districts, schools, and students, while convenience sampling was applied for qualitative data. Multivariable logistic regression assessed factors influencing parental acceptance of COVID-19 vaccination, and qualitative analysis identified barriers and facilitators.

**Results:**

A total of 2017 parents participated in the study. The overall parental acceptance to vaccinate their children against COVID-19 was 76.4%. Multivariate logistic regression analysis showed that parents who were literate (*p* = 0.004), not vaccinated against COVID-19 (*p* = 0.012), had less than or equal to four family members (*p* < 0.001) and a history of COVID-19 infection in the family (*p* = 0.036) were less likely to accept the COVID-19 vaccine for their children. Key barriers to vaccination included uncertainty over the protection provided by the vaccine, fear about side effects, and misconceptions about the vaccine whereas belief in the vaccine, perceived severity of COVID-19 disease, and bundling with routine vaccination were the key facilitators.

**Conclusion:**

These findings highlight the importance of increasing adult COVID-19 vaccination. Developing policies focusing on parents with higher literacy, staying in smaller families, and previous COVID-19 infection among family members will help to increase the vaccine uptake among children. Interventions for the integration of these vaccines with routine immunization or availability at schools may help in increasing COVID-19 vaccine acceptance.

## 1 Introduction

The COVID-19 pandemic, driven by the novel coronavirus SARS-CoV-2, has significantly disrupted societies and healthcare systems globally since its emergence in late 2019 (1). Amidst efforts to curb the spread of virus, the development, approval, and distribution of effective vaccines have become pivotal in the global response.

Among the many challenges and complexities related to the COVID-19 vaccine rollout, one particularly significant aspect involves the acceptance of these vaccines among parents of children between the ages of 6 and 12 years. Children and adolescents typically experience less severe illness and lower mortality rates from SARS-CoV-2 compared to adults (2). However, children and adolescents are still susceptible to SARS-CoV-2 infection and can transmit the virus to others, with the risk of infection and transmission increasing with age (3). During the Omicron variant surge in 2022, reported COVID-19 cases among children saw a dramatic spike, coinciding with the relaxation of public health and social measures in most countries. COVID-19 outbreaks have been observed in secondary schools, summer camps, and day-care centers, especially in instances where neither physical distancing nor mask-wearing was implemented to mitigate transmission risks (4).

While studies on booster dose acceptance among adults, such as one conducted in Indonesia, highlight changing attitudes toward vaccination over time, similar studies focusing on parents’ willingness to vaccinate their children remain limited (5). Since parents rely on healthcare providers for vaccine-related guidance, the challenges faced by professionals in managing pandemic-related stress and misinformation may further impact parental decision-making regarding childhood COVID-19 vaccination. (6) As of December 6, 2023, India has administered a total of 2,20,67,78,090 vaccine doses. Among these, 6,21,62,331 first doses and 5,38,04,027 s doses have been administered to adolescents aged 15–18 years. Additionally, over 4.13 million first doses and around 3.25 million second doses have been administered to the age group 12–14 years (7).

For children under the age of 12 years, parents play a crucial role in deciding on whether they will receive a COVID-19 vaccine. Understanding the factors that influence parental decision-making in this age group is crucial for designing effective public health campaigns, vaccination strategies, and interventions to achieve widespread immunization and build herd immunity. The decisions parents make regarding their children’s COVID-19 vaccination are not only pertinent to the health and well-being of the individual child but also to broader public health goals. So, this mixed method study intended to assess COVID-19 vaccine acceptance and the barriers and facilitators to acceptance among parents across different regions of India before the vaccine rollout for children in the country.

## 2 Methodology

### 2.1 Study settings

The present mixed-method study using convergent approach was conducted from March to September 2023 among parents from five geographical regions (North, South, East, West and North-east) in India to explore acceptance to vaccinate their children between 6 and 12 years against COVID-19. One state from each zone was selected namely Delhi, Kerala, Bihar, Maharashtra and Assam and the study was implemented in two districts from each state.

#### 2.1.1 Ethics statement

The study was approved by the Institutional Ethics committee (IEC) of ICMR-National AIDS Research Institute with registration number NARI/EC/2023/668.

### 2.2 Quantitative methods (cross-sectional)

#### 2.2.1 Sample size and sampling

The sample size was calculated using the formula: *n* = z^2^pq/d^2^ at 95% confidence level, 5% absolute precision and 5% non-response rate. A total of 1880 participants (376 from each of the 5 states) across five zones of India was calculated for the study (8). Accordingly, a total of 2017 participants were included from all study sites of which, 1925 and 92 were parents of school going and non-school going children, respectively. Multistage sampling was used to select districts, schools, and students.

#### 2.2.2 Selection of schools

From each state, a systematic listing and mapping of government and private primary and secondary schools was done in the selected districts with the help of education departments. Five schools from each district were then chosen randomly for inclusion in the study.

#### 2.2.3 Study participants

A complete roster of students from each selected school was compiled, encompassing all children aged between 6 and 12 years in grades one through five. Six to seven students representing various class divisions were randomly selected from each class, amounting to a total of 35–36 students per school. Parents of the selected children were subsequently contacted and invited to participate in the study until the predetermined sample size was attained. One parent from each participating family, either the mother or father, willing to give informed consent was included in the study.

Additionally, data collection was conducted among 20 parents from each state whose children were not enrolled in school, considering that approximately 5.37% of children of primary school age do not attend school (9).

#### 2.2.4 Study tool development and validation

The study questionnaire underwent a rigorous development process, beginning with input from experts in epidemiology, paediatrics, education, non-governmental organizations, and community health. Initial drafts were subjected to validation by these experts, with subsequent modifications based on their feedback. Following validation, the questionnaire was translated into the local languages of the five states by independent translators.

To ensure face validity, a pilot study involving 15 parents was conducted, and adjustments to the questionnaire were made based on their feedback. This iterative process ensured that the final questionnaire was comprehensive and culturally appropriate for the study population.

#### 2.2.5 Study questionnaire

The questionnaire comprised six sections designed to capture comprehensive information from the participants:

1. Sociodemographic: This section recorded details about the parents and their children, including age, marital status, education, monthly income, relationship with the child, number of children, age, and gender of children, presence of any chronic disease among children, history of COVID-19 infection in the family, and total number of doses of COVID-19 taken by family members.
2. Knowledge, Attitude, and Practices toward COVID-19 vaccination: The second section included questions assessing parental knowledge, attitudes, and practices regarding COVID-19 vaccination. It covered topics such as vaccination awareness, parental attitudes toward vaccinating their child, and practices related to COVID-19 vaccination.
3. Routine Immunization: This section encompassed questions related to routine immunization practices for the participants’ children.
4. COVID-19 vaccination among Parents: The fourth section collected information on COVID-19 vaccination among parents, including the total number of doses received, type of vaccine, perceptions of vaccination benefits, reasons for missing doses, and experiences of side effects.
5. COVID-19 vaccination among adolescent family members: Similar to section four, this section gathered information on COVID-19 vaccination among adolescent family members.
6. Vaccine acceptance: The final section documented parents’ willingness to vaccinate their child against COVID-19, their preferences regarding the type and place of vaccination, and any reasons for reluctance to vaccinate their child.

#### 2.2.6 Data analysis

The demographic and clinical characteristics of study participants were analyzed using descriptive statistics such as median and interquartile range (IQR), means and standard deviations for continuous variables and proportions for categorical variables. Parental vaccine acceptance served as the outcome variable. We analyzed through binary logistic regression to examine the association with independent variables. Significant variables at the 5% level were further analyzed using multivariate logistic regression to identify factors associated with vaccine acceptance. Data analysis was performed using SPSS 20.0 (SPSS, Inc.; Chicago, IL, USA).

### 2.3 Qualitative methods

#### 2.3.1 Study settings

All in-depth interviews were conducted across all five study sites in person ensuring that interviewees were thoroughly briefed about the study objectives. Prior to participation, interviewees provided written informed consent. To maintain anonymity and confidentiality, each participant was assigned a unique code based on their state of residence.

#### 2.3.2 Participants and sampling

This qualitative study encompassed in-depth interviews conducted across diverse regions of India, strategically selected from five geographical zones: North, South, West, East, and Northeast (Delhi, Kerala, Maharashtra, Bihar, and Assam). Eligible participants were either father or mother of children aged 6–12 years, fluent in the study languages (Marathi, Hindi, Malayalam, Assamese), and willing to provide written informed consent. Recruitment utilized convenience sampling, predominantly approaching parents at schools, with additional outreach to settlements and workplaces for parents of non-school-going children. A total of 24 parents were invited to participate in face-to-face in-depth interviews.

#### 2.3.3 Interview guide for in-depth interviews

The interview guide was meticulously crafted through an extensive literature review and collaborative discussions among site investigators, drawing upon the expertise of various professionals including teachers, sociologists, paediatricians, counsellors, and representative parents. A workshop was conducted to review and finalize the guide, incorporating inputs from local contexts provided by site investigators. It was translated and validated in regional languages (Marathi, Hindi, Assamese and Malayalam) and English to ensure accessibility and cultural relevance (Supplementary material 1).

The guide, piloted and revised for consistency, served as a flexible tool for prompting discussions. It commenced with open-ended inquiries about parents’ experiences with routine immunization and COVID-19 vaccines, gradually delving into factors influencing vaccine acceptance for their children aged 6–12 years. Key domains covered included: (a) Parents’ experiences with routine immunization and COVID-19 vaccination, (b) Perceived acceptance of COVID-19 vaccine for their children, (c) Perceived barriers and facilitators for vaccination, and (d) Preferences regarding vaccination site, cost, and mode of administration.

Discussions were conducted in the local language to ensure comprehension, with the interviewer providing explanations for any unclear questions using simpler terms. All interviews were digitally recorded for accurate documentation.

#### 2.3.4 Data saturation

Data saturation was achieved across all study sites, as evidenced by the consistent themes observed in the final set of in-depth interviews. To ensure rigor and minimize bias, a total of 8 select transcribed interviews were reviewed by other study team members for validation and confirmation of findings. This process helped enhance the credibility and reliability of the study outcomes.

#### 2.3.5 Data analysis

Data analysis commenced after the completion of data collection, with data saturation assessed during the collection period as in-depth interviews (IDIs) were conducted in a shorter timeframe. The audio recordings were transcribed verbatim and translated into English by the study team and an experienced translator. Two qualitative researchers (SP & PS) independently familiarized themselves with the study data by repeatedly reading the transcripts, followed by coding the interviews into potential themes and subthemes. Initial manual coding was followed by importing the transcripts into N-Vivo 14 (QSR) for further analysis. Discrepancies in coding were resolved by referring to the original transcripts and reaching consensus during daily study team meetings. The investigators engaged in deliberate discussions to identify emerging categories and themes, which were then refined and reviewed before sharing the final themes within the research team.

##### 2.3.5.1 Content validity and pilot testing of study tools

A panel of experts from different fields reviewed the items for clarity, relevance, and comprehensiveness, and revisions were made accordingly. Reliability was assessed qualitatively by piloting the questionnaire with a small group of participants to ensure clarity and consistency in responses. To enhance clarity and usability, the instrument was revised after pilot study, with simplified terms, refined responses, addition of local dialect, and improved formatting. The results were discussed in the internal meetings and review session with investigators. Qualitative feedback, common challenges, and field observations were analyzed to assess participant engagement and comprehension.

## 3 Results

### 3.1 Quantitative

A total of 2017 study participants were included in the study. Out of the total, 1925 (95.4%) parents of school-going children aged 6–12 years participated in the study. Among them, 1,153 (59.9%) belonged to government schools, while 772 (40.1%) were from private schools. Additionally, 92 (4.6%) parents of non-school-going children were also included in the study.

#### 3.1.1 Demographic characteristics of study participants

The mean age of all 2017 study participants was 35 years (SD = 6.2; range: 19–65; IQR: 30–39). Among them, 70% (1409) were females. Majority of the participants, 47.9% (966) had education up to 10th standard. On average, the family size was 4.6 (SD = 1.2), with 14.7% reporting a history of COVID-19 infection. Additionally, 91.2% of families had received at least one dose of the COVID-19 vaccine.

#### 3.1.2 State wise COVID-19 vaccine acceptance for their children among study participants

The study found an overall acceptance rate of approximately 76.4% among parents of children aged between 6 and 12 years for the COVID-19 vaccine. Among the states, Bihar and Delhi exhibited the highest acceptance rates at 96 and 93%, respectively. In Maharashtra (70%) and Kerala (69%), more than two-thirds of the participants were willing to vaccinate their children in this age group. Half of the participants from Assam (49%), expressed willingness for COVID-19 vaccination for their children.

The overall vaccine acceptance among parents of children going to public schools was 81% (905/1123) as compared to those whose children went to private schools, 71% (507/710). (Table 1).

**Table 1:** State wise, school wise Vaccine acceptance among study participants for their children (6–12 years of age).

	COVID-19 vaccine acceptance among parents of 6–12 years children	
States	Public School	Private school
Maharashtra	160/204 (78%)	108/179 (60%)
Kerala	174/225 (77%)	75/135 (56%)
Assam	103/206 (50%)	81/133 (61%)
Delhi	233/240 (97%)	137/156 (88%)
Bihar	235/248 (95%)	106/107 (99%)

#### 3.1.3 Factors associated with COVID-19 vaccine acceptance among study participants for their children between 6 and 12 years of age

##### 3.1.3.1 Univariate logistic regression analysis

The analysis revealed several significant factors influencing the acceptance of COVID-19 vaccination among parents. Mothers were significantly less likely to vaccinate their children compared to fathers [Odds Ratio (OR): 0.77, 95% Confidence Interval (CI): 0.61–0.97, *p*-value = 0.024]. Conversely, parents with employment showed a significantly higher acceptance of the vaccine compared to those without employment (OR: 1.27, 95% CI: 1.03–1.56, *p*-value = 0.025). Additionally, literate participants were less likely to accept vaccination for their children compared to others (OR: 0.58, 95% CI: 0.41–0.83, *p*-value = 0.003).

Furthermore, participants from families with less than or equal to four members and those with a history of COVID-19 infection in the family were less likely to accept vaccination for their children compared to others (OR: 0.66, 95% CI: 0.53–0.81, *p*-value <0.001 and OR: 0.74, 95% CI: 0.56–0.97, *p*-value = 0.029, respectively). Participants who had received at least one dose of the COVID-19 vaccine themselves were more likely to vaccinate their children (OR: 2.59, 95% CI: 1.62–4.12, *p*-value <0.001).

Additionally, participants who did not experience any side effects from the COVID-19 vaccine were significantly more likely to accept vaccinating their children compared to those who did experience side effects (OR: 1.35, 95% CI: 1.08–1.68, *p* = 0.007). Moreover, participants with vaccinated adolescent siblings in the family and those with all family members having received at least one dose of the vaccine were more likely to accept COVID-19 vaccination for their children (OR: 3.45, 95% CI: 2.20–5.40, *p*-value <0.001 and OR: 1.65, 95% CI: 1.19–2.30, *p*-value = 0.003, respectively). Age, type of school, and routine immunization were not found to be significantly associated with the acceptance of COVID-19 vaccine.

##### 3.1.3.2 Multivariate logistic regression

The multivariate logistic regression analysis revealed some significant factors influencing COVID-19 vaccination acceptance among participants. Specifically, participants who were literate, had smaller family sizes, and reported a history of COVID-19 infection in the family were significantly less likely to accept COVID-19 vaccination for their children. Conversely, participants who had received at least one dose of COVID-19 vaccination themselves were found to be two times more likely to vaccinate their children compared to those who had not (Adjusted Odds Ratio: 2.26, 95% Confidence Interval: 1.20–4.28, *p*-value: 0.012) (Table 2).

**Table 2:** Factors associated with COVID-19 vaccine acceptance among study participants for their children between 6 and 12 years of age.

Characteristics	Total (*n* = 2017)	COVID-19 vaccine acceptance Yes (*n* = 1,540, 76.4%)	COVID-19 vaccine acceptance No (*n* = 477, 23.6%)	Univariate logistic regression OR (95% CI)	*p* value	Multivariate logistic regression AOR (95% CI)	*p* value
Age of the participants (years)							
≤ 35	1,167 (57.9)	886 (75.9)	281 (24.1)	0.95 (0.77–1.16)	0.595	__	__
>35	850 (42.1)	654 (76.9)	196 (23.1)	1			
Sex of the parents							
Female	1,409 (69.9)	1,056 (74.9)	353 (25.1)	0.77 (0.61–0.97)	0.024	0.80 (0.58–1.09)	0.159
Male	608 (30.1)	484 (79.6)	124 (20.4)	1		1	
Status of school for children							
School	1925 (95.4)	1,470 (76.4)	455 (23.6)	1.02 (0.62–1.66)	0.951	__	__
Non school	92 (4.6)	70 (76.1)	22 (23.9)	1			
Type of school							
Government	1,153 (57.2)	915 (79.4)	238 (20.6)	1.21 (0.73–1.99)	0.458	__	__
Private	772 (38.3)	555 (71.9)	217 (28.1)	0.80 (0.49–1.33)	0.396	__	__
Non school	92 (4.6)	70 (76.1)	22 (23.9)	1			
Education of participants							
Literate	1767 (87.6)	1,330 (75.3)	437 (24.7)	0.58 (0.41–0.83)	0.003	0.58 (0.40–0.84)	0.004
Illiterate	250 (12.4)	210 (84.0)	40 (16.0)	1		1	
Employment of participants							
Employed	991 (49.1)	778 (78.5)	213 (21.5)	1.27 (1.03–1.56)	0.025	1.13 (0.85–1.49)	0.398
Unemployed	1,026 (50.9)	762 (74.3)	264 (25.7)	1		1	
History of COVID-19 infection in the family							
Yes	297 (14.7)	212 (71.4)	85 (28.6)	0.74 (0.56–0.97)	0.029	0.74 (0.56–0.98)	0.036
No	1720 (85.3)	1,328 (77.2)	392 (22.8)	1		1	
Number of family members							
≤4	1,099 (54.5)	802 (73.0)	297 (27.0)	0.66 (0.53–0.81)	<0.001	0.66 (0.53–0.81)	<0.001
>4	918 (45.5)	738 (80.4)	180 (19.6)	1		1	
Routine immunization of the children							
Yes	1964 (97.4)	1,496 (76.2)	468 (23.8)	0.65 (0.32–1.35)	0.250	__	__
No	53 (2.6)	44 (83.0)	9 (17.0)	1		__	__
Participants with at least one dose of COVID-19 vaccine							
Yes	1941 (96.2)	1,497 (77.1)	444 (22.9)	2.59 (1.62–4.12)	<0.001	2.26 (1.20–4.28)	0.012
No	76 (3.8)	43 (56.6)	33 (43.4)	1		1	
Participants having any side effect after COVID-19 vaccine (*n* = 1941)							
No	793 (40.9)	636 (80.2)	157 (19.8)	1.35 (1.08–1.68)	0.007	__	__
Yes	1,148 (59.1)	861 (75.0)	287 (25.0)	1			
COVID-19 vaccination among adolescents in the family (*n* = 528)							
Yes	317 (60.0)	280 (88.3)	37 (11.7)	3.45 (2.20–5.40)	<0.001	__	__
No	211 (40.0)	145 (68.7)	66 (31.3)	1			
Uptake of at least one dose of COVID-19 vaccine by all members in the family							
Yes	1840 (91.2)	1,421 (77.2)	419 (22.8)	1.65 (1.19–2.30)	0.003	1.32 (0.83–2.10)	0.235
No	177 (8.8)	119 (67.2)	58 (32.8)	1		1	

#### 3.1.4 Knowledge, attitude and practices related to COVID-19 vaccine for children among parents

The majority of participants (95%) were aware of the availability of the COVID-19 vaccine in the country. They demonstrated good knowledge regarding the vaccine’s protective effects (85.6%), and lowering the risk of death (82%). Participants also had awareness of potential side effects of the vaccine (81.1%). However, understanding that the vaccine can be taken even after a previous infection (53.6%) or that one could still contract COVID-19 post-vaccination was relatively lower (60.2%). A significant portion of parents perceived the vaccine as curative (59.7%) and safe and beneficial for children (68.1%). Additionally, 69.2% were willing to recommend vaccination to friends and family. However, very few had received a booster dose. Of the total participants, 48.3% of parents were aware of the need to register their children on the COWIN software application for vaccination. Despite this, participants exhibited good practices such as consulting healthcare workers for vaccine-related queries (92.2%), verifying news authenticity with healthcare professionals before believing it (86.9%), and explaining vaccines to their children prior to vaccination (87.7%).

### 3.2 Qualitative results

A total of 24 IDIs were conducted from five different study sites across India. Basic demographic details from these participants were collected. Of these, 13 were males and 11 females, with a median age of 35 years. IDIs were conducted among 17 parents of school-going children and 7 parents of non-school-going children. Most of the parents were educated till 12^th^ standard and above and few were illiterate. The duration of the IDIs ranged between 30 min and one hour.

#### 3.2.1 Facilitators

##### 3.2.1.1 Belief in ‘vaccine’

The majority of interviewees expressed confidence in the vaccines administered through the National Routine Immunization Program for children and emphasized the pivotal role of vaccines in safeguarding against various diseases.

“Vaccine is meant to increase immunity as well as to protect those (children) from infection.” (Mother of a school-going child, Kerala)

“What … vaccines are for child’s own health … In future child should not get any disease for that … for precaution-vaccines should be given.”

(Father of school going child, Bihar)

###### 3.2.1.1.1 Perceived severity of COVID-19 disease

A significant number of participants shared personal anecdotes and perspectives on the severity and potential complications of COVID-19. Motivated by a desire to prevent such outcomes, they expressed willingness to have their children vaccinated against the disease.

“Everyone looked at it (COVID-19) fearfully. We were affected. Had tough times. Nobody came to our house. Sort of isolated that’s what scared us more. Nobody comes home or rather we cannot go to anyone … we had severe physical issues. We don’t want this to happen with our kids.”

(Mother of a school-going child, Kerala)

“Yes, there was a lot of fear in the mind. I was continuously moving. I had to visit customer’s residence. There was immense fear of COVID and its complications. That’s why, I think that our kids must be vaccinated against COVID.”

(Father of school going child, Maharashtra)

###### 3.2.1.1.2 Vaccine confidence/protection from disease

Several parents shared their first-hand experiences with COVID-19 infection, noting that they had relatively mild symptoms and few complications, which they attributed to the protection afforded by the vaccine.

“We ourselves are the motivators, aren’t we? We have taken the vaccine. COVID-19, waves have come 2-2 times, 3-3 times Sir. We have not had it (COVID-infection)”

(Father of a non-school-going child, Bihar)

“In India, the death rate has reduced, and I have not heard of any deaths due to COVID-19 in the past year. Therefore, I believe that vaccination has played a significant role in reducing the death rate.”

(Mother of school going child, Assam)

###### 3.2.1.1.3 Creating awareness through social media/community leader

Several interviewees emphasized the importance of raising awareness about the availability of COVID-19 vaccines for young children.

“Social media is very fast. It is super-fast … unknowingly it affects … it should be used extensively … that’s what I think. A message can be sent to any person just sitting idle … It is of great advantage. “

(Father of a school-going child, Bihar)

“Yes, even after going door to door and like polio vaccination, if it is spread in the same way, then it will be effective.”

(Father of a school-going child, Bihar)

##### 3.2.1.2 Role of motivators

###### 3.2.1.2.1 Community leaders

“Apart from social media, the influential leaders are there. They have also a big role to play.”(Father of a non-school-going child, Bihar)

###### 3.2.1.2.2 Schoolteachers

“Many parents are such that they listen to teachers a lot. If every teacher informs every parent to give this vaccine, they will surely listen.”

(Father of a school-going child, Maharashtra)

###### 3.2.1.2.3 Health care workers and doctors

“Everyone-like people working for Corona, social workers used to give information like ASHA workers. They used to come around us and tell us. They can be of help if you want to increase the acceptance.”

(Father of a school-going child, Delhi)

##### 3.2.1.3 Bundling with the routine vaccination

Many interviewees also suggested the convenience of integrating COVID-19 vaccination with the routine government immunization program.

“When immunization card is given, we follow as per the card-which dose must be given when … it has helped us immensely. Immunization has been done neatly due to this. If it (COVID vaccination) has been included in it (routine immunization) and made compulsory … after some days … everyone will take and if a child enters in that age group, they (parents) will give.”

(Mother of a school-going child, Maharashtra)

“See that would be very much easy for parents, because they will say this has to be done,like the flu, like the tetanus like the any other this things, that has to be also a part, It will be important, If it is included in the routine, then that also important, it will be easy to follow …”

(Mother of school going child, Delhi)

##### 3.2.1.4 Trust in government healthcare and guidelines

The majority of parents expressed trust in government healthcare and COVID-19-related guidelines.

“Children’s parents should understand that everything is beneficial for children whatever the government is doing.”

(Mother of a school-going child, Delhi)

“We don’t follow such media. They might carry anything. We follow government guidelines.”

(Mother of school going child, Kerala)

#### 3.2.2 Barriers

##### 3.2.2.1 Uncertainty over protection provided by the vaccine

Some parents voiced their uncertainty regarding the effectiveness of the vaccine in providing protection.

“Because there were people, who were infected even after the vaccine. So, don’t believe that vaccines will prevent us from getting infected.”

(Mother of school-going child, Kerala)

“Even after vaccination, I became positive (infected). My husband had even taken booster dose but still he got infected because of me. Earlier he had taken first dose of Covishield, then also he got positive. Report of his staff was positive then he got infected then he took booster dose and got infected after it (booster dose) and I also had taken two doses … even after that I became positive (infected).”

(Mother of school going child, Maharashtra)

###### 3.2.2.1.1 Fear about side effects

All parents expressed a common concern about the potential side effects of the COVID-19 vaccine on children.

“How can I give the vaccine now that they are small? Now that he is young, who will take the risk if something happens to him?”

(Father of a non-school-going child, Assam)

“I don’t think it is necessary. I mean, the older ones had these issues (side effects), what if they give it (vaccine) to the younger children for Covid-19. They have a future.”

(Father of school going child, Delhi)

##### 3.2.2.2 Misconceptions/rumours about the vaccine

Numerous parents harbored various misconceptions regarding the COVID-19 vaccine and its potential effects.

“Because some of the adults died after receiving the injections the children would also die. I think that’s it.”

(Mother of non-school-going child, Assam)

“Many people say that after getting the vaccine, they feel weak and have pain in their legs. They feel tired.” (Mother of a school-going child, Assam)

##### 3.2.2.3 Lack of faith in the vaccination program

Some participants expressed concerns about the conduct of the routine immunization program, questioning whether the rollout of COVID-19 vaccination for their children would encounter similar challenges.

“My husband’s parents were not interested in giving routine vaccinations to my kids. It’s my own decision to vaccinate a child with routine vaccinations as I fear that something may happen in the future if I do not vaccinate him. They don’t believe in vaccination.”

(Mother of school-going child, Kerala)

“Even if it (vaccination) is made compulsory, we will have to take a bit of experience … if we have a bit of experience … at least 10% information if we have about any harm after vaccination … we will have a slight idea … our concept gets cleared … because no one in the government informs us about side effects … no one tells … they just give injections. the way we do counselling and take consent … it does not happen when it (vaccination) is made compulsory … how will we come to know what our kid will suffer from.”

(Mother of school going child, Maharashtra)

##### 3.2.2.4 Lack of awareness

A minority of parents also cited a lack of information about the COVID-19 disease and the vaccine as a factor influencing their decision-making.

“If a parent is not coming. How will parents get informed? So obviously that is creating a barrier. Like this is not for my kids. Many of the parents are not aware of the COVID-19 disease and its vaccines.”

(Mother of a school-going child, Delhi)

##### 3.2.2.5 Complacency due to a decrease in number of cases

A small number of parents did not perceive the severity of COVID-19 at present, pointing out the decrease in reported cases and the absence of new cases in the country.

“But until now, it doesn’t seem urgent according to the current situation. The current situation suggests that COVID’s impact is decreasing, so the urgency for COVID dose is decreasing.”

(Father of a school-going child, Maharashtra)

“Now case proportions have been reduced. I have also not taken booster dose. Now it has happened that we have become secured. There is not much risk left now … that has happened now.”

(Father of non-school going child, Maharashtra)

**Figure 1:**
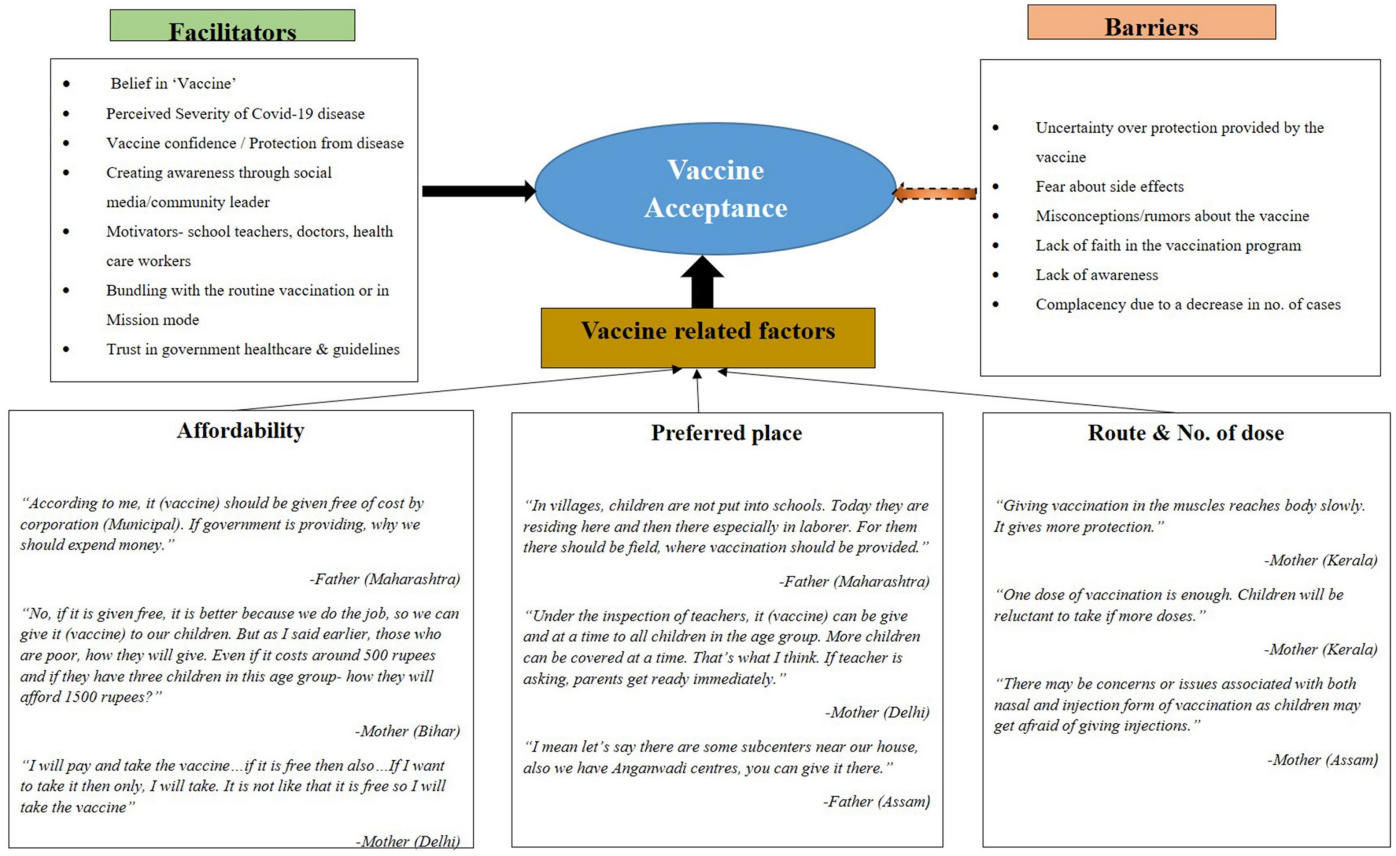
Facilitators and Barriers among parents to vaccination against COVID-19 in the pediatric population.

Figure 1 serves as a visual representation of the significant facilitators and barriers identified across different categories and themes during the qualitative analysis. Moreover, it succinctly encapsulates key vaccine-related factors, such as affordability, accessibility, and considerations regarding routes and dosages. This comprehensive summary provides valuable insights into the complexities surrounding the topic at hand, facilitating a deeper understanding and informed decision-making.

## 4 Discussion

Our study provides data on parental acceptance of COVID-19 vaccination across diverse regions of India and offers information for designing effective vaccination strategies. By examining vaccine acceptance within different cultural contexts, the present study enhances our understanding of regional factors influencing parental attitudes toward vaccination. Overall, our findings contribute significantly to the understanding of parental COVID-19 vaccine acceptance enabling the development of more targeted and effective vaccination initiatives.

The overall parental vaccine acceptance rate was 76.4%, with regional variations. These differences may stem from disparities in healthcare infrastructure, cultural beliefs, prior exposure to COVID-19, and trust in government initiatives. The observed acceptance rate in this study was lower than the 84% reported in China (10) and the 85% reported in another Indian study (11), which may be because of the evolving perceptions of vaccine necessity due to decreased infection rates. A systematic review estimated global parental acceptance at 57% (12), suggesting that India’s relatively high rate could be attributed to government-led immunization campaigns and past experiences with mass vaccination programs.

An inverse association between literacy and vaccine acceptance was observed, which aligns with findings from Hong Kong (13) and Latin America (14). In these settings, higher education levels were associated with increased exposure to conflicting information, leading to concerns over vaccine safety. Literate parents may have greater access to online sources, including misinformation, which can heighten skepticism. Addressing these concerns through transparent, science-based communication from trusted sources such as pediatricians and educators can help counteract misinformation and build vaccine confidence.

Parental acceptance was lower among those with a history of COVID-19 infection in the family or smaller family sizes. Parents who had experienced COVID-19 may perceive natural immunity as sufficient, reducing the urgency for vaccination. In contrast, a study from northwestern India found higher acceptance among families affected by COVID-19 (15), possibly due to firsthand experiences of severe illness reinforcing the perceived benefits of vaccination. A study from Qatar suggests that parents of adolescents previously infected with COVID-19 were more likely to be hesitant about vaccination (16). These differences highlight the role of individual experiences and risk perception in shaping vaccine decisions. Additionally, smaller families might feel less vulnerable to infection or prioritize caution regarding potential vaccine side effects, reflecting findings that risk perception influences vaccine hesitancy.

Our study suggests that smaller families may perceive lower COVID-19 transmission risks due to assumed immunity from past infections. Additionally, smaller families may adopt more cautious behaviors to avoid exposure to potential vaccine side effects. Furthermore, our study highlighted a positive correlation between parental vaccination status and their willingness to vaccinate their children, consistent with research from the United States (17). Similarly, a systematic review emphasized the importance of parental vaccination status as a key predictor of their children’s vaccination intentions (18).

While parents had generally good knowledge of vaccine benefits, misconceptions persisted, emphasizing the need for targeted educational interventions. A longitudinal study from the United States demonstrated that vaccine perceptions evolve over time, influenced by emerging data and public discourse (19). This suggests that sustained communication efforts, particularly those addressing safety concerns, are essential to maintaining vaccine confidence. Misconceptions about vaccine side effects or necessity must be actively countered through tailored messaging that reinforces scientific evidence and public health recommendations.

This study underscores the pivotal role of teachers and pediatricians in advocating for COVID-19 vaccination among children, as they were identified as the most reliable sources of information for parents. Additionally, healthcare workers, community leaders, and social media were found to be instrumental in promoting COVID-19 vaccine acceptance, consistent with findings from previous studies (20, 21). However, parents cautioned that these influencers and media platforms must possess accurate understanding and information about COVID-19 vaccines to effectively motivate the parent community. Failure to do so could lead to the dissemination of fake news, misinformation, or rumors, eroding trust in vaccines and impacting parental willingness to vaccinate their children, as similarly observed in a study from Turkey (22). In the current era of infodemics, it is imperative to avoid negative messaging and instead provide clear, concise information about vaccines to dispel misunderstandings and foster trust in vaccination efforts. The present study highlights the significant influence of individual experiences related to COVID-19 in enhancing vaccine acceptance, substantiating findings from other studies (23, 24). The emotional and financial hardships endured by families throughout the pandemic, is noteworthy that some parents opted to adopt a wait-and-see approach, preferring to learn from the experiences of their peers and family members, as evidenced in a study conducted in India (25). The voices of parents sharing their personal encounters with COVID-19 and vaccination could serve as a vital strategy to instill confidence in the vaccine among hesitant individuals.

Our study identified key barriers to COVID-19 vaccination, including concerns about side effects, distrust in the vaccination program, and hesitancy due to the vaccine’s rapid development. Parental concerns about side effects likely arise from the rapid vaccine rollout and evolving information on safety, which created uncertainty. Distrust in the vaccination program may be influenced by misinformation, past negative healthcare experiences, or a lack of transparent communication from authorities. Additionally, declining COVID-19 cases may have led to a reduced perceived risk, making vaccination seem less urgent. Similar concerns have been documented in other studies, emphasizing the need for targeted public health messaging to address parental fears and improve trust in vaccination (15, 26, 27).

In our study, parents preferred schools, immunization camps, and government health centers for their children’s vaccinations, mainly due to convenience, cost-free services, and trust in these institutions. This preference might be based on the prior research in India, where government-led immunization programs have historically contributed to high vaccine coverage. Ensuring continued accessibility to free vaccination services at trusted locations can help sustain vaccine uptake (15, 23, 28).

To enhance vaccine uptake, specific strategies should be prioritized. School-based vaccination programs provide a structured and trusted environment for immunization, reducing logistical barriers. Mobile clinics can increase accessibility for underserved communities, particularly in rural and peri-urban areas. Training healthcare workers to address vaccine-related concerns ensures that parents receive accurate information from trusted professionals. Community leaders can be engaged in awareness campaigns to foster social acceptance. Social media fact-checking initiatives can counter misinformation, and door-to-door outreach programs can provide direct engagement opportunities with hesitant parents. Implementing these targeted strategies can help overcome vaccine hesitancy and improve uptake.

This study has some limitations. The findings may not be fully generalizable due to regional differences in healthcare access and vaccine perceptions, which can shape attitudes differently across states. The cross-sectional design limits causal interpretations, meaning the identified associations do not imply direct cause-and-effect relationships. Additionally, responses may have been influenced by the specific COVID-19 situation at the time, which may not reflect future attitudes as the pandemic evolves. Interviewing only one parent per household may not fully capture shared decision-making dynamics, and potential biases in sampling and social desirability responses should be considered. Future studies should employ more representative sampling methods and longitudinal designs to track changes in vaccine acceptance over time.

## 5 Conclusion

The study provides insights into parental acceptance rates and associated factors regarding the COVID-19 vaccine in India, aiding policymakers in devising strategies to enhance vaccine uptake among children. Tailored interventions, educational materials, and vaccination campaigns should consider family dynamics, literacy levels, prior COVID-19 exposure, and cultural variations across states. Encouraging parental vaccination can positively impact children’s vaccine uptake. Key facilitators for acceptance include belief in vaccines, perceived severity of COVID-19, confidence in vaccine efficacy, and awareness efforts via social media or community leaders. Conversely, barriers include concerns about side effects, vaccine misconceptions, distrust in vaccination programs, and complacency due to declining COVID-19 cases. Implementing vaccination drives in schools or health centers or integrating COVID-19 vaccines into routine immunization programs, can address parental concerns. Strategies balancing perceived costs, community awareness, and vaccine integration with routine immunization can help overcome barriers to vaccination.

## Data Availability

The original contributions presented in the study are included in the article/Supplementary material, further inquiries can be directed to the corresponding author.
