# Supplementary material for: COVID-19 vaccine acceptance and associated factors among parents of children between 6 and 12 years: a multicenter mixed method study in India
Source: Front Public Health. 2025 Jun 25;13:1513419. doi: 10.3389/fpubh.2025.1513419 (PMC12237963; doi:10.3389/fpubh.2025.1513419)
Supplement: Supplementary file 1 [file Table_1.DOCX]

| **COVID-19 vaccine acceptance among parents of 6-12 years old children in India: A mixed method study: IN DEPTH INTERVIEW GUIDE** |
| --- |
| **IDENTIFICATION AND CONSENT STATUS** |
| State Code : |
| District Code : |
| School / Non -school going Code : |
| IDI Number : |
| Student ID :  State code District School/ Non School going IDI Number  Code Code |
| Type of School : Government Private |
| Date of interview: _______________ |
| Name of the interviewer: ____________________________________ |
| Parent contact status : Available Not available |
| Consent status : Agreed Refused |
| Informant : Mother Father |

“Can I seek your permission to switch on the tape recorder for recording our conversations so that I don’t miss out on anything and play it later to listen to our conversations and learn from them?

(ENCIRCLE BELOW THE RESPONSE FOR RECORDING AS APPROPRIATE)

1. Yes
2. No

**Start Recorder if permitted and Read the introduction**:

My name is _________________ (Name of the moderator). I will conduct your interview and keep notes in addition to recording your responses.

Before I begin, I would appreciate it, if you could please share something about yourself

- How old are you? -______Age in completed years)
- Have you ever attended school? --------------
- Which class did you pass in school? -----------------
- What do you do for a living? _______
- How much money do you make in a month (in Rupees)? ___________
- How long have you been in the place you currently live in? _______
- No family members ___________________
- Children ___________________

We are interested in knowing your views about issues that will be asked. Please note that there is no wrong or right response. If there are any apprehensions that you would like to talk about you may bring them up even if we haven’t asked them. If there is any question that you would like to ask I would take note of them and answer them after this interview. If we are unable to answer any of the questions, we would refer them to an appropriate person.

As mentioned earlier, the main goal of this discussion is to understand the acceptability of Covid-19 vaccination among parents of children of 6-12 years of age. I want to remind you that what we discuss here will be kept confidential and that I will not share your personal information or responses with anyone outside the study. Shall we start our discussion now?

(ENCIRCLE BELOW THE RESPONSE FOR RECORDING AS APPROPRIATE)

1. Yes
2. No

If he says NO may I know your reasons for declining ________________________?

__________________________________________________________________________________________________________________________________________________________________

Thank you for your opinion

| **IDI Guide** |
| --- |
| 1. **How comfortable were you in accepting my invitation for participation in this study?**   *Probes*   - Did you feel it was important for you to participate? Why? - What benefits do you see of participating? Why? |
| 1. **Where do you currently go to seek health care services?**   *Probes*  Which facility do you go to? Why?  Please elaborate the reasons for preferring this facility / these facilities  --------------------------------------------------------------------------------------------------------------------------------------------   1. **Do you believe in the concept of vaccination/ vaccine? Yes/ No**   *Probes*  If No- why?  Have your children completed their routine immunization as per their age? Yes/ No  If No- why?  ------------------------------------------------------------------------------------------------------------------------------------------   1. **What is your opinion about the current covid -19 scenario in the country?**   *Probe*  Caseload  Different vaccines available  Different strains  ---------------------------------------------------------------------------------------------------------------------------------------   1. **Experience with COVID-19 vaccination**   Have you ever suffered from Covid-19 -Yes/No?  5a) What are your views/opinions about the Covid-19 vaccination program/vaccine?  *Probe*  Was it available?  Was it accessible?  Was it convenient?  5b) Have you taken a vaccine against Covid-19?  *Probe*  If ‘yes’, why?  5c) Which one?  *Probe*  Why this vaccine only?  5d) How many dosages?  5e) What motivated you to go for vaccination?  *Probe*  Media  Fear  Community leader  Any other  5f) Where did you take the vaccine?  *Probe*  Govt centre/ private/any other setting  Why a particular centre?    5g) Why did you take only one dose (Ask only if one dose has been taken by the participant)?  5h) Did you take a precautionary dose?  *Probe*  If yes, why?  If no, why?  5i) If the vaccine is not taken at all, may I know the reasons?  *Probe*  Any fear  Anxiety  Apprehension  Concerns  Any previous personal/ family/ community experience  5j) What was your feelings/experience when the first dose was taken?  *Probe*  Did you suffer from any side effect?  5k) What about other family members? Have they been vaccinated?  *Probe*  Any side effects in them?  5l) Who takes the decision for the vaccination in your family?  5m) Whether same vaccine was taken by other family members or different?  ------------------------------------------------------------------------------------------------------------------------------------------   1. **What do you think about the necessity of these vaccines against covid-19 for children?**   --------------------------------------------------------------------------------------------------------------------------------------------   1. **Are you aware of availability of vaccines against covid-19 for children?**   *Probe*  Source of information  --------------------------------------------------------------------------------------------------------------------------------------------   1. **Will you be comfortable to get your children vaccinated?**   *Probe*  If yes, why  If no, why?  Any fear  Anxiety  Apprehension  Concerns  Any previous personal/ family/ community experience  -------------------------------------------------------------------------------------------------------------------------------------------   1. **What benefits of vaccinating your child do you foresee?**   --------------------------------------------------------------------------------------------------------------------------------------------   1. **What risks after vaccinating your child against covid-19 do you foresee?**   **-------------------------------------------------------------------------------------------------------------------------------------------**   1. **What is your opinion on making vaccination of children compulsory?**   -------------------------------------------------------------------------------------------------------------------------------------------   1. **What can be the major motivators for paediatric covid -19 vaccination and rank them based on importance?**   *Probe*  Please specify the reason  Role of Personal/ family/ community  Healthcare system and health workers  Socio-cultural  Environmental  Health system/ institutional  Economic or political factors  --------------------------------------------------------------------------------------------------------------------------------------------   1. **What can be the major barriers to the paediatric covid -19 vaccination and rank them based on importance?.**   *Probe*  Please specify the reason  Role of Personal/ family/ community  Healthcare system and health workers  Socio-cultural  Environmental  Health system/ institutional,  Economic or political factors  -------------------------------------------------------------------------------------------------------------------------------------------   1. **What do you think about the influence of the social media on paediatric vaccination?**   *Probe*  Positive effect  Negative effect  --------------------------------------------------------------------------------------------------------------------------------------------   1. **Any influence of community leaders on covid-19 paediatric vaccination?**   *Probe*  Positive effect  Negative effect  --------------------------------------------------------------------------------------------------------------------------------------------   1. **What would be the role of routine immunization program for Covid vaccination in children?**   --------------------------------------------------------------------------------------------------------------------------------------------   1. **What are the common misconceptions that the community has regarding the COVID-19 vaccine in children?**   *Probe*  What measures are taken to address them / can be taken to prevent them  --------------------------------------------------------------------------------------------------------------------------------------------   1. **Mode of covid-19 vaccine administration in children**   *Probe*  Which route do you prefer for your children?  Oral/Nasal/Injection  Why?  -------------------------------------------------------------------------------------------------------------------------------------------   1. **Covid-19 vaccination schedule & place**   *Probe*  What should be the schedule?  How should it be given-as a routine programme mode or mass vaccination compaign?  Where should it be given?    In schools  Private clinic  Govt center  Please give me the reason for selecting a particular facility  ***--------------------------------------------------------------------------------------------------------------------------------------------***   1. **Costs-Covid-19 vaccine**   *Probe*    How should it be given – free of cost or at some cost?  Why free    Why cost?    What should be the cost if made available  --------------------------------------------------------------------------------------------------------------------------------------------   1. **Explain in detail- What can be done to further improve the uptake of a paediatric vaccine against Covid-19?**   --------------------------------------------------------------------------------------------------------------------------------------------   1. **Any other point that you think has not been discussed in this interview**   --------------------------------------------------------------------------------------------------------------------------------------------   1. **Please give your general recommendations/ suggestions that you think will improve the paediatric vaccine uptake in the future**     Thank You! |

**Extra Notes**
